# Echocardiographic Changes in Chronic Kidney Disease Patients on Maintenance Hemodialysis

**DOI:** 10.7759/cureus.8969

**Published:** 2020-07-02

**Authors:** Farah Anum Jameel, Abdul Mannan Junejo, Qurat ul ain Khan, Sudeep Date, Ahmad Faraz, Syed Hasan Mustafa Rizvi, Fatima Ahmad, Muhammad Tahir

**Affiliations:** 1 Nephrology, Jinnah Postgraduate Medical Center, Karachi, PAK; 2 Orthopaedics and Trauma, Cumberland Infirmary, Carlisle, GBR; 3 Trauma and Orthopaedics, Leeds Teaching Hospitals NHS Trust, Leeds, GBR; 4 Internal Medicine, Peterborough City Hospital, Peterborough, GBR; 5 Anaesthesia, Punjab Institute of Cardiology, Lahore, PAK; 6 Orthopaedics, Jinnah Postgraduate Medical Center, Karachi, PAK

**Keywords:** chronic kidney disease, maintenance hemodialysis, echocardiography

## Abstract

Introduction

Chronic kidney disease (CKD) carries a significant association with cardiac diseases, which suggests a minor reduction in the glomerular filtration rate (GFR) can act as an independent risk factor for causing cardiovascular abnormalities. Patients of CKD having cardiovascular disease (CVD) had three to thirty times higher risk of mortality as compared to the general population. In addition, mortality among cardiovascular patients has been found to be twofold higher in CKD stage 2 patients and three-fold higher in patients with stage 3 CKD, when collated to patients with normal renal function. Furthermore, cardiomyopathy among hemodialysis (HD) is due to the presence of coronary artery obstruction, reduction in coronary reserves, and left ventricular (LV) physiological-structural abnormalities secondary volume and pressure overload. Echocardiography is a gold standard diagnostic modality for the identification of cardiac structural and functional abnormalities. Therefore, the evaluation of echocardiographic parameters in patients of CKD can help to determine the risk and prognosis of CVD in patients of CKD. In the present study, we evaluated the echocardiographic findings in patients of CKD on maintenance hemodialysis.

Methods

This cross-sectional study was conducted in the nephrology unit of Jinnah Postgraduate Medical Center between March 2019 to October 2019. A total of 100 patients who were on maintenance for more than one year were included in the analysis. Two-dimensional transthoracic echocardiography was done in each patient for the determination of cardiac structural and functional parameters such as LV hypertrophy, LV systolic dysfunction, and LV diastolic dysfunction.

Results

The mean age of the patients was 46.9±12.8 years. There was male dominance with male/female ratio 63/37. There were 39% hypertensive and 62% anemic patients. LV dysfunction was diagnosed in 31% of patients, LV diastolic dysfunction in 47% patients, and left ventricular hypertrophy (LVH) in 55% of patients. LVH was found in 74.3% hypertensive patients versus only 42.6% non-hypertensive patients (p-value 0.001). LV systolic dysfunction was also high in hypertensive patients, 46.1% versus 21.3% patients in non-hypertensive patients (p-value 0.008).

Conclusion

There is a high frequency of cardiac functional and structural abnormalities in CKD patients on HD especially in patients having concomitant hypertension. LVH is the most common structural defect and LV diastolic dysfunction is the most common functional cardiac defect in CKD patients on hemodialysis.

## Introduction

Chronic kidney disease (CKD) carries a significant association with cardiac diseases, which suggests a minor reduction in the glomerular filtration rate (GFR) is an independent risk factor for causing cardiovascular abnormalities [1]. Manjunath et al. reported that patients with GFR ≤59 mL/min/1.73 m2 have a 38% higher risk of cardiovascular disease (CVD) development as compared to those having GFR ≥90 mL/min/1.73 m2 [1]. CVD is associated with >50% of the deaths in CKD patients. Patients of CKD having CVD had three to thirty times higher risk of mortality as compared to the general population [2].In addition, mortality among cardiovascular patients has been found to be twofold higher in CKD stage 2 patients and three-fold higher in patients with stage 3 CKD, when collated to patients with normal renal function [3].

Left ventricular hypertrophy (LVH) is one of the common structural cardiac defects in CKD patients. LVH significantly increases the risk of cardiac ischemia, heart failure, and is a strong predictor of mortality in CKD patients [4]. LV dysfunction is an initial precursor of CVD and leads to LVH in the follow-up period [5]. Furthermore, cardiomyopathy among hemodialysis (HD) is due to the presence of coronary artery obstruction, reduction in coronary reserves, and left ventricular physiological-structural abnormalities secondary volume and pressure overload [6]. When efforts to reduced left cardiac preload are not made, adaptation in left ventricular is activated, which leads to a decrease in capillary density, diastolic dysfunction, and disturbances in intraventricular conduction, dilatation, and more compensatory hypertrophy [7].

These phenomena increase vulnerability to increase electrical excitability, leading to sudden cardiac death among these patients [8]. Echocardiography is a gold standard diagnostic modality for the determination of cardiac structural and functional abnormalities. Therefore, the evaluation of echocardiographic parameters in patients of CKD can help to determine the risk and prognosis of CVD in patients of CKD [9]. In the present study, we evaluated the echocardiographic findings in patients of CKD on maintenance HD.

## Materials and methods

This cross-sectional study was conducted in the nephrology unit of Jinnah Postgraduate Medical Center within 07 months duration from March 2019 to October 2019. A total of 100 patients who were on maintenance for more than one year were included in the analysis. Patients who were already known for having CVD such as coronary artery disease, valvular heart disease congenital heart, or primary cardiomyopathy were excluded. Approvalafromahospital ethical committee was taken. Written consent for participation of the study was also signed by each patient.

In all patient's baseline laboratory investigations such as complete blood count, renal parameters, lipid profile were measured in each patient as a routine investigation protocol. Two-dimensional echocardiography was done in each patient for the determination of cardiac structural and functional parameters. Patients having LV ejection fraction (EF) <50% were diagnosed with having LV dysfunction. For determination of LV diastolic dysfunction, the E/A ratio was calculated using Doppler velocity measurements, E/A ratio <0.75, or >1.8 was labeled as LV diastolic dysfunction. Patients having an intraventricular thickness or LVaposterior wall thickness ≥12mm were diagnosed as having LV hypertrophy(LVH).

Already known cases of hypertension or patients having BP ≥140/90 were labeled as hypertensive. While anemia was diagnosed if the hemoglobin level was <135 gm/dL in male patients and <12.5 gm/dL in female patients.

Data were analyzed in SPSS version 23.0 (IBM, Armonk, USA). The Pearson Chi-square test was applied to determine the association of anemia and hypertension with cardiac functional and structural abnormalities. p-Value ≤0.05 was taken as significant. 

## Results

The mean age of patients was 46.9±12.8 years. There was male dominance with male/female ratio 63/37. There were 39% hypertensive and 62% anemic patients (Table 1). 

**Table 1:** Baseline Characteristics of Study Participants

Variable	Value
Mean Age	46.9±12.8 (Range; 21-82)
Gender (Male/Female)	63/37
Serum Creatinine (mg/dL)	5.36±1.49
Diabetes Mellitus	32%
Hypertension	39%
Anemia	62%

**Figure 1:**
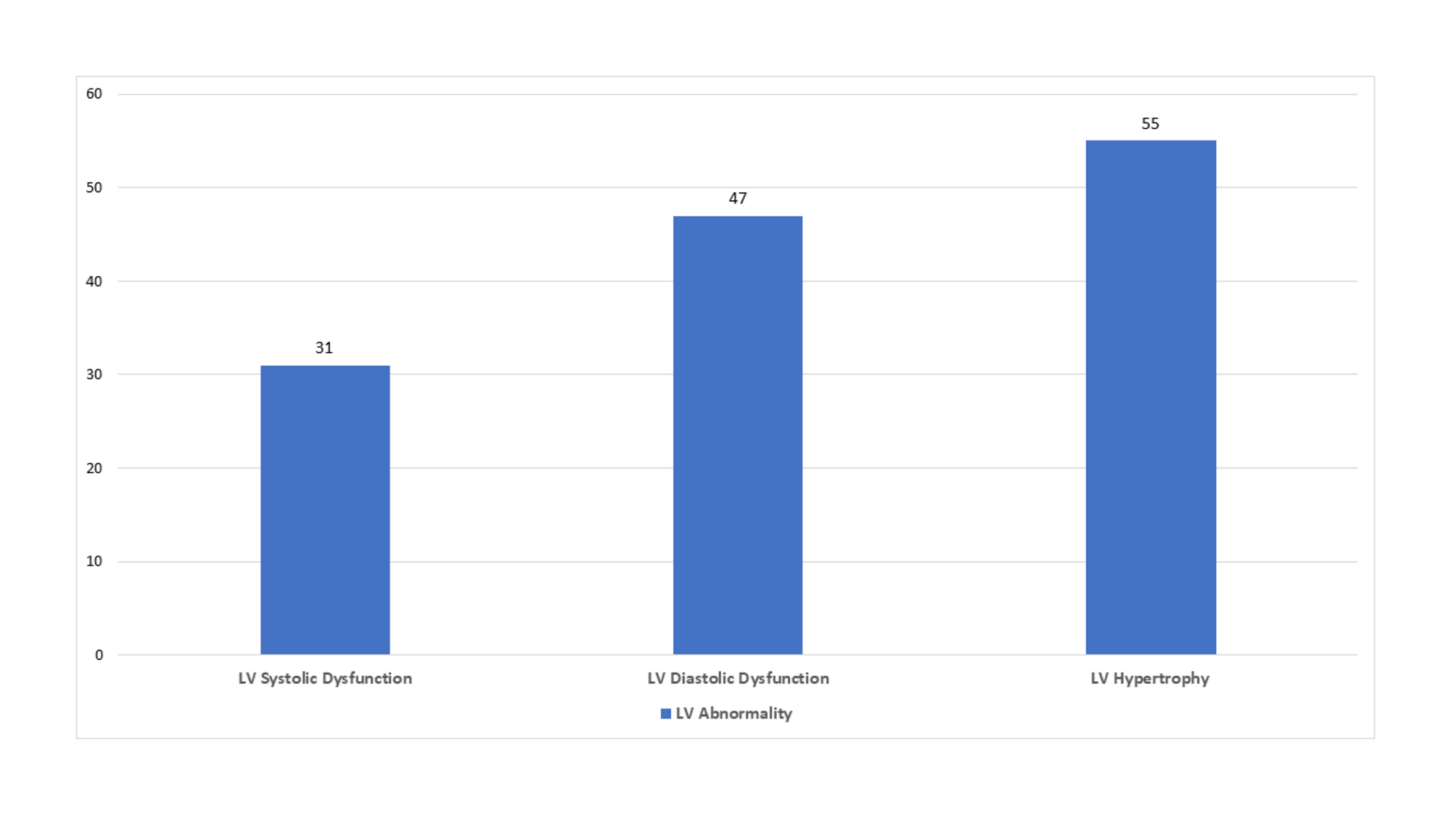
Echocardiographic Abnormalities in patients of CKD CKD- Chronic Kidney Disease, LV- Left Ventricle

Regarding echocardiographic abnormalities, LV dysfunction was diagnosed in 31% patients, LV diastolic dysfunction in 47% patients and LVH in 55% of the patients (Figure 1).

There was higher frequency of LV systolic dysfunction and LVH in patients having hypertension as compared to non-hypertensive patients (p-value 0.008 & 0.001 respectively) (Table 2).

**Table 2:** Comparison of Echocardiographic Abnormalities in Hypertensive and Non-Hypertensive Patients LV- left ventricular, LVH- left ventricular hypertrophy

	Hypertension (N=39)	Non-Hypertension (N=61)	p-Value
LV Systolic Dysfunction	18 (46.1%)	13 (21.3%)	0.008
LV Diastolic Dysfunction	22 (56.4%)	25 (40.9%)	0.13
LVH	29 (74.3%)	26 (42.6%)	0.001

## Discussion

CKD patients have higher proportions of congestive heart failure that is associated with a higher mortality rate in these patients [5]. Echocardiography is a valuable tool to assess the assess changes in function and structure of the heart that result from CKD. Abnormal LV geometry, reduction in interventricular septum strength, and changes in LV mass index are important parameters that are affected by CKD in patients with preserved EF [6].

Previous studies have reported anemia, volume overload, electrolyte abnormalities edema, and hypertension as risk factors that alter the risk of CVD in CKD patients [7,8]. In the present study, anemia was diagnosed in 62.0% of patients, hypertension in 39%, and diabetes mellitus in 32% of patients. A study by Tsilonis et al. reported diabetes mellitus in 24% of patients and hypertension in 22% of patients of CKD patients on HD [9].

The current study reports the most common cardiac abnormality was LVH, found in 55% of patients, followed by LV diastolic dysfunction in 47% patients and LV systolic dysfunction in 31% of patients. A study conducted by Shivendra et al. reported LVH in 48% of patients, diastolic dysfunction in 51.42% patients, and systolic dysfunction in 28.57% patients of CKD on maintenance HD [10]. Agarwal et al. reported LV diastolic dysfunction in 53.2% patients and LV systolic dysfunction in 30% of patients having severe CKD [4]. Another study by Laddha et al. reported LVH in 74.3% patients, LV diastolic dysfunction in 61.4% patients, and systolic dysfunction in 24.3% patients [11]. A similar study by Ahmed et al. LVH in 80% of patients, LV diastolic dysfunction in 53.3% patients, and LV systolic dysfunction in 36.3% patients [12]. 

Some studies have reported LV systolic dysfunction in all patients of HD [13,14] which is very high as compared to our study and the above-mentioned studies. The possible reason for this high proportion may be that these studies used the positron emission tomography scan for determination of systolic dysfunction that uses contrast-induced ischemic changes for diagnosis of ischemia and is superior to echocardiography for determination of cardiac dysfunction [14].

In our study, hypertension was found a significant risk factor for cardiac abnormalities. In our study, LVH was found in 74.3% hypertensive patients versus in only 42.6% non-hypertensive patients. Similarly, LV systolic dysfunction was also high in hypertensive patients, 46.1% versus 21.3% patients in non-hypertensive patients. Barre et al. also reported similar findings; they reported LVH in 64.47% hypertensive patients and in 33.3% non-hypertensive patients (20) [3]. Shivendra et al. also reported similar findings; they reported LVH in 51% hypertensive versus in only 8.57% normotensive patients (21) [10].

In summary, cardiac abnormalities are common in patients of CKD on maintenance HD. So echocardiography should be performed at regular intervals in these patients especially those having hypertension along with CKD. A timely diagnosis of cardiac abnormalities can help in the early management of these complications and can help to reduce cardiac induced morbidity and mortality in CKD patients.

## Conclusions

In conclusion, structural and physiological abnormalities of right and left ventricle are more commonly affected among patients with mild or moderate renal disorder, whereas right ventricle function and structure are independently associated with CKD progression. Furthermore, there is higher frequency of cardiac abnormalities among CKD patients on maintenance HD especially in patients having concomitant hypertension. LVH is the most common structural defect and LV diastolic dysfunction is the most common functional cardiac defect in CKD patients on HD. Additional workup of cardiovascular pharmacological management specifically among the HD group is required to make an evidence-based clinical decision to alleviate CVD in this higher risk group. Technology advances in HD may provide an opportunity to prevent CVD associated with dialysis treatment.​
